# Linkage of individual-patient data confirm protection of prophylactic human papillomavirus vaccination against invasive cervical cancer

**DOI:** 10.1093/jnci/djae042

**Published:** 2024-03-19

**Authors:** Marc Arbyn, Pegah Rousta, Laia Bruni, Lina Schollin Ask, Partha Basu

**Affiliations:** Unit of Cancer Epidemiology, Belgian Cancer Centre, Scientific Institute of Public Health, Brussels, Belgium; Department of Human Structure and Repair, Faculty of Medicine and Health Sciences, University Ghent, Ghent, Belgium; Unit of Cancer Epidemiology, Belgian Cancer Centre, Scientific Institute of Public Health, Brussels, Belgium; Cancer Epidemiology Research Program, Catalan Institute of Oncology–IDIBELL, L’Hospitalet de Llobregat, Barcelona, Spain; Centro de Investigación Biomédica en Red: Epidemiología y Salud Pública (CIBERESP CB06/02/0073), Madrid, Spain; Unit for Vaccination Programmes, Public Health Agency of Sweden, Stockholm, Sweden; Department of Women’s and Children’s Health, Karolinska Institutet, Stockholm, Sweden; International Agency for Research on Cancer, Lyon, France

The Scottish study of Palmer et al., published in the current issue of this journal, linking vaccination files with cervical cancer screening data and the national cancer registry, demonstrates excellent protection against invasive cervical cancer among girls immunized at the age of 12 to 13 years with the bivalent human papillomavirus (HPV) vaccine (Cervarix, GSK, Rixensart, Belgium) (1). The study completes and strengthens the evidence of the high level of effectiveness of primary cervical cancer prevention by HPV vaccination based on intervention trials and population-based surveillance of real-world data built up over the last two decades.

## Efficacy and effectiveness of HPV vaccines in terms of prevention of infection and cervical precancer

Reduction of the burden of invasive cervical cancer, although the main purpose of HPV vaccination, was not an endpoint of the randomized HPV vaccination trials that led to their licensing and introduction of HPV vaccines in many countries. Having cancer as an outcome would have required very costly and lengthy observation periods and postpone the availability of vaccines for decades. Therefore, the World Health Organization (WHO) had recommended reduction of cervical intraepithelial neoplasia (CIN) of grade 2 or CIN3 or worse, associated with the HPV types targeted by the vaccine, as the first trial outcome (2). Moreover, international experts invited by the International Agency of Research of Cancer and the National Cancer Institute, in 2014, agreed that persistent type-specific HPV infection, determined by validated assays, may be an acceptable endpoint for future prophylactic HPV vaccine trials, recognizing the strong causal link between persistent infection and cervical cancer development (3,4).

Randomized trials evaluating the bi-valent (Cervarix), the quadri- and nona-valent (Gardasil and Gardasil9, Whitehouse Station, NJ, USA) HPV vaccines have shown long-lasting immunogenicity and excellent protection against persistent infection with the HPV vaccine types, certain cross-reacting types, and associated cervical precancer (5-8). Vaccine efficacy (VE) was higher than 90% in teenagers and women younger than 26 years who were HPV DNA negative at enrollment. VE was lower but still substantial (∼50%) among all vaccinated women, irrespective of initial HPV status (9). These data suggest a very high level of protection among young teenagers (mainly nonexposed to HPV), who are the main target of routine vaccination, and a moderate but significant protection among older teenagers and young adults (many of whom having initiated sexual contacts) who are the target of catchup vaccination programs. However, among women older than 25 years, the protection against cervical intraepithelial neoplasia (CIN) of grade 2 or 3 or worse due to whatever HPV type was low to absent (9-11).

Meta-analyses of real-world data confirmed the evidence of HPV vaccine effectiveness against infection with HPV16 and 18, against cross-reacting HPV31, 33, and 45 and associated cervical precancerous lesions. Protection was excellent when HPV vaccines are administered before the age of 20 and low when administered at older ages (12).

## Effectiveness of HPV vaccines in terms of prevention of invasive cervical cancer

**Figure 1.**
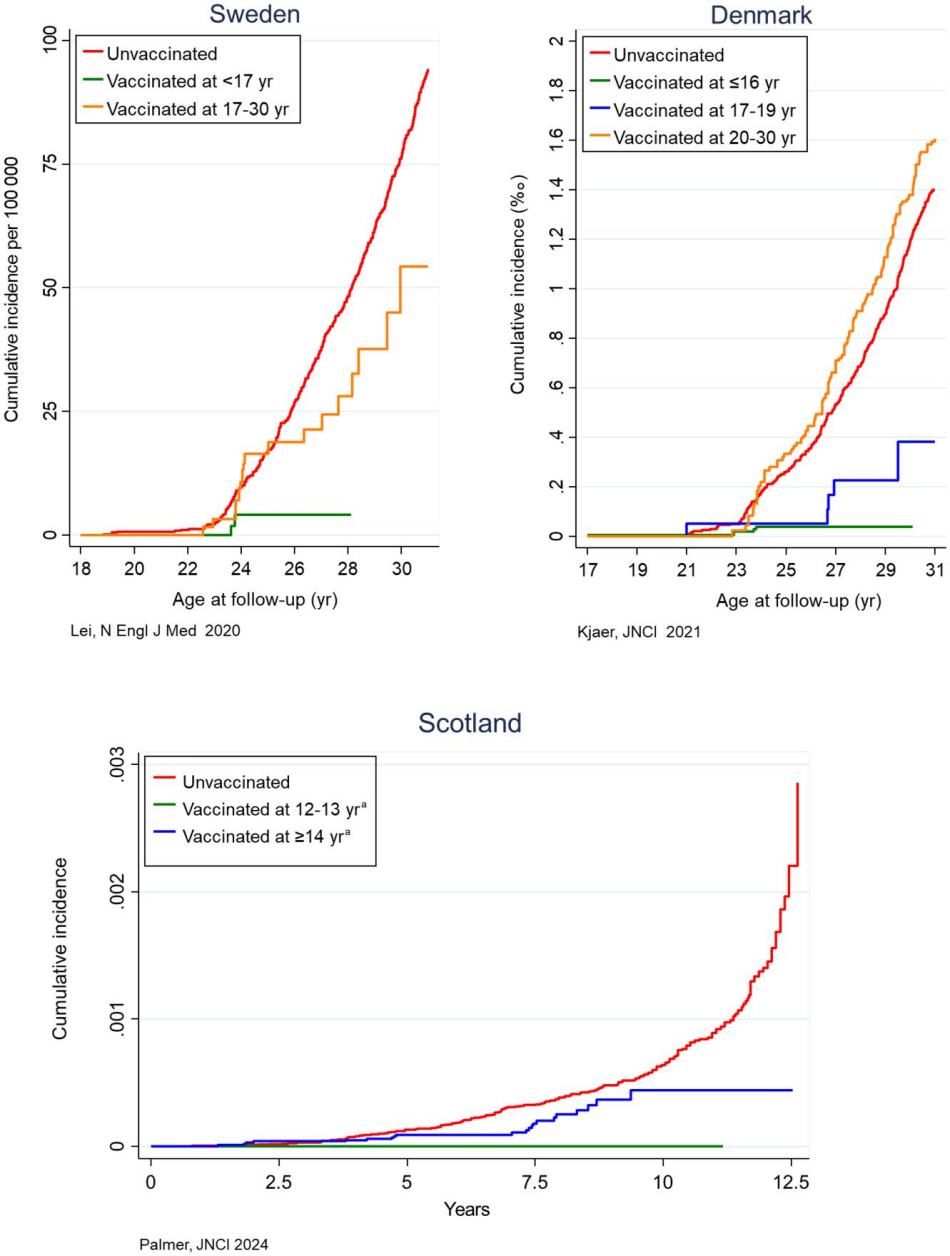
Cumulative incidence of cervical cancer stratified by human papillomavirus vaccination status and age at vaccination (see legend), observed in linkage studies conducted in Sweden (14), Denmark (15), and Scotland (1), joining individual patient data from vaccination and cancer registries. The X-axis in the two plots on top expresses the years at follow-up, whereas the plot at the bottom expresses the years since start of screening invitation. ^a^For Scotland, restricted to subjects who were completely vaccinated.

Conservative epidemiologists have criticized surrogate evidence based on protection against precursors, arguing that HPV infection and cervical precancerous lesions usually clear spontaneously and that by treating screen-detected lesions, progression to cancer can be avoided (5,13). However, three recent linkage studies, similar to the one of Palmer et al., conducted in Sweden (14), Denmark (15), and England (16), have demonstrated effectiveness against cervical cancer by vaccinating with Gardasil (Table 1, Figure 1). Moreover, a Finish monitoring study, enrolling vaccinated cohorts from trials and nonvaccinated subjects linked to the national cancer registry, also yielded excellent protection against cervical cancer (*P* = .03) (17).

Figure 1 and Table 1 replicate the cumulative incidence of cervical cancer and estimated vaccination effectiveness scanned from the four recently published linkage studies. The green curves involving the youngest participants at vaccination are consistently located near the bottom of the plots, reflecting a very high effectiveness (Table 1). Among Scottish girls who were vaccinated at the age of 12 to 13 years, irrespective of the number of doses, the incidence was zero (effectiveness of 100%). At older ages, cancer protection became progressively lower (blue or orange curves approximating the red curve for nonvaccinated females). The Danish data showed no difference in cancer incidence between subjects vaccinated after the age of 20 (orange curve) and nonvaccinated subjects. It should be noted that vaccine effectiveness estimated from the four linkage studies were adjusted for various socioeconomic, demographic, time, age, and other factors. However, bias due to residual confounding, inherent to observational data, cannot be excluded.

**Table 1.** HPV vaccine effectiveness estimated from four linkage studies joining individual records from vaccination databases with cancer registries

Reference	Age at	VE	Cofactors adjusted for
(Country)	vaccination (years)	(95% CI)	adjusted for
Lei, 2020 (14)	<17	81% (35% to 95%)	Age, county, calendar year, birth country of mother, education and income of parents, occurrence of (pre)cancer in mother
(Sweden)	17-30	36% (0% to 61%)	
Kjaer, 2021 (15)	≤16	87% (59% to 96%)	VE adjusted for attained age and education of parent(s)
(Denmark)	17-19	69% (−7% to 91%)	
	20-30	−14% (-49% to 13%)	
Falcaro, 2021 (16)	12-13	87% (72% to 94%)	VE adjusted for age-cohort interactions, screening campaign and J Goody campaign^b^ effect
(England)	14-16	62% (52% to 71%)	
	16-18	34% (25% to 41%)	
Palmer, 2024 (1)	12-13^a^	100% (67% to 100%)	VE adjusted for deprivation index
(Scotland)	14-18^a^	69% (54% to 79%)	

a Completely vaccinated (2 doses at least 5 months apart or 3 doses).  CI = confidence interval; VE = vaccine effectiveness as reported or computed (VE = risk ratio − 1).

b Publicity surrounding celebrity Jade Goody, who died from cervical cancer.

Unfortunately, the Scottish and three other linkage studies cannot be pooled in an overall statistic because of different metrics, scales, and age categories. This will be addressed within *metaSURV*, a statistical project aiming for pooling of longitudinal data using digitized Kaplan-Meier curves.

These findings underpin recommendations to vaccinate teenagers as the first target group in routine vaccination programs, to offer vaccination of older teenagers and young adults in transitory catchup vaccination activities, depending on local cost-effectiveness, and to preserve vaccination of women aged 25-30 or older according to individual clinical shared information (6,18-20).

## Importance of linkage studies, legal and administrative barriers

Linkage of individual patient data (IPD), although a powerful tool for monitoring the impact of vaccination and other preventive interventions, is hampered tremendously by legal restrictions (such as the General Data Protection Regulation in Europe). Nordic countries and the United Kingdom have found administrative solutions to perform linkages of pseudonymized records, albeit still with limitations (for instance, restrictions in reporting small data cells, impeding more precise age groupings), and offered templates to be followed by other countries. Ministers responsible for Health and Justice should create legal frameworks facilitating monitoring of preventive health programs, including linkage of IPD. This challenge is currently being addressed within the Europe’s Beating Cancer Plan^†^ through several projects such as the Joint Action PERCH (PartnERship to Contrast HPV, www.projectperch.eu) (21). To integrate primary and secondary prevention of HPV-related diseases, comprehensive registries of IPD data and linkages between them are fundamental for running preventive programs (targeted invitations, precise determination of coverage rates, fine-tuning of screening policies adjusted to vaccination status, present HPV genotype and prior screening history), increase in quality and efficiency of cancer prevention and evaluation of these programs. Moreover, linking this type of population data enables the answering of scientific questions that cannot be addressed through trials such as protection against other HPV-related cancers; short- and long-term impact of alternative dosages and other HPV vaccines; determination of upper age bench marks for prophylactic HPV vaccination (22); occurrence of breakthrough HPV infections and type-replacement among vaccinated subjects; long-term safety by linkage with obstetrical and morbidity registries). As authors of this invited editorial, we strongly recommend international cross-border compilation of IPD that may increase statistical power and overcome the limitations of small cell censoring highlighted by Palmer et al. This may provide the granularity to better determine crucial aspects such as the optimal catchup maximum age, vaccine scheduling, and risk-based subgroup strategies.

The paper of Palmer et al. (1), in line with other similar linkage studies, nicely completed the evidence base on the effectiveness of prophylactic HPV. They all show that HPV vaccination of young teenage girls provides a very high protection against cervical cancer irrespective of the number of doses. The protection decreases by age, but vaccination of older adolescents is still substantial. International agencies should support pooling of multicountry-linked IPD data to increase the speed and statistical power to address currently unanswered questions, which are crucial for reaching WHO’s cervical cancer elimination initiative.

† https://health.ec.europa.eu/system/files/2022-02/eu_cancer-plan_en_0.pdf

## Data Availability

Not applicable.
